# Supplementary material for: Regulation between LRRK2 and PP2A signaling in cellular models of Parkinson’s disease
Source: Biochem J. 2026 Jun 26;483(6):1097–114. doi: 10.1042/BCJ20260194 (PMC13212363; doi:10.1042/BCJ20260194)
Supplement: Supplementary Figures S1-S6 [file BCJ-2026-0194_supp.pdf]

Supplementary Figures for:

Regulation between LRRK2 and PP2A signaling in cellular models of Parkinson's disease

Panagiotis S. Athanasopoulos[1], Anna Memou[2], Franz Y. Ho[1], Ahmed Soliman[1,3], Henderikus Pots[1], Vassiliki Papadopoulou[2], Felix von Zweydorf[4,5], Saiganesh Sriraman[1], Alexander Marc Thouin[1], Laurine Vandewynckel[6], William Sibran[6], Marie-Christine Chartier-Harlin[6], R. Jeremy Nichols[7], Elisa Greggio[8,9], Jean-Marc Taymans[6], Christian Johannes Gloeckner[4,5], Hardy J. Rideout[2*], and Arjan Kortholt[1,10*]

[1]Department of Cell Biochemistry, University of Groningen, Nijenborgh 7, 9747 AG, Groningen, The Netherlands

[2]Center for Clinical, Experimental Surgery, and Translational Research; Biomedical Research Foundation of the Academy of Athens; Athens, Greece

[3]Division of Engineering in Medicine, Department of Medicine, Brigham Women's Hospital, Harvard Medical School, Boston, MA 02115, USA

[4]German Center for Neurodegenerative Diseases, 72076 Tübingen, Germany

[5]Institute for Ophthalmic Research, Center for Ophthalmology, University of Tübingen, Tübingen, Germany

[6]Univ. Lille, Inserm, CHU Lille, UMR-S 1172 - LilNCog - Lille Neuroscience & Cognition, F-59000 Lille, France.

[7] Department of Structural Biology, St Jud Children's Research Hospital, Memphis TN, USA

[8]University of Padova, Department of Biology, Padova, Italy

[9]Centro Studi per la Neurodegenerazione (CESNE), University of Padova, Italy

[10]YETEM-Innovative Technologies Application and Research Centre Suleyman Demirel University West Campus, Isparta, Turkey

*Corresponding authors:

Arjan Kortholt, Nijenborgh 7, 9747 AG, Groningen, The Netherlands, a.kortholt@rug.nl

Hardy J. Rideout, BRFAA, Soranou Efessiou 4, 115 27, Athens, Greece, hrideout@bioacademy.gr

Short title: LRRK2/PP2A signaling in Parkinson's disease

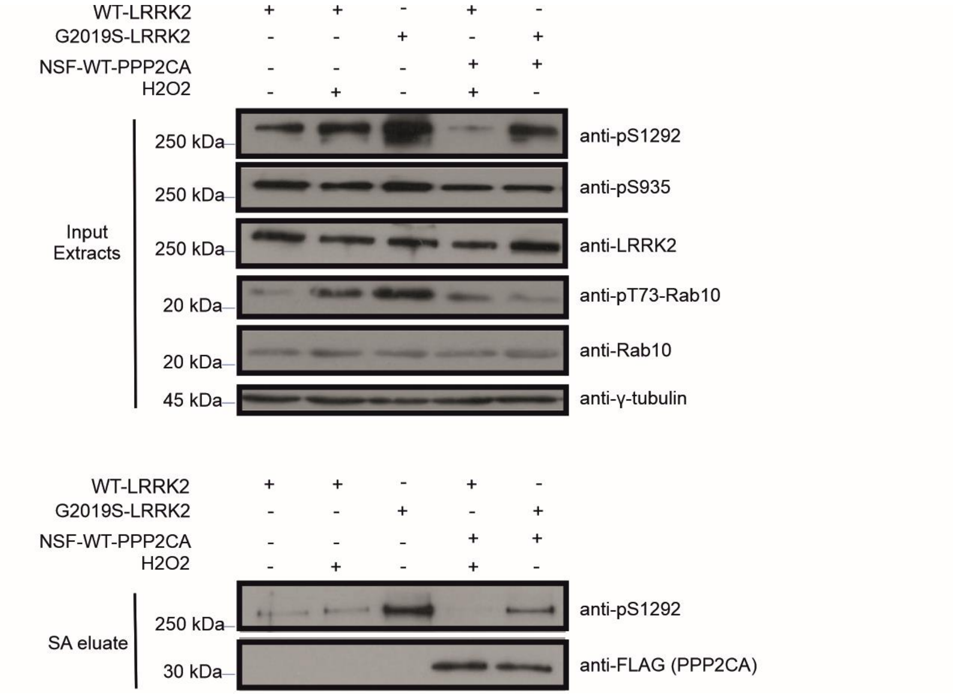

Supplementary Fig. 1: Overexpression of PPP2CA in cells reduces the kinase activity of LRRK2. Co-expression of PPP2CA reduces cellular kinase activity of LRRK2 in HEK293T cells, as assessed by auto-phosphorylation at Ser1292 in both total cell extracts as well as isolated dimers (SA eluate, lower panel), and phosphorylation of Rab10 (T73). Additionally, note that PPP2CA co-elutes with dimeric LRRK2 purified on SA-coated resin.

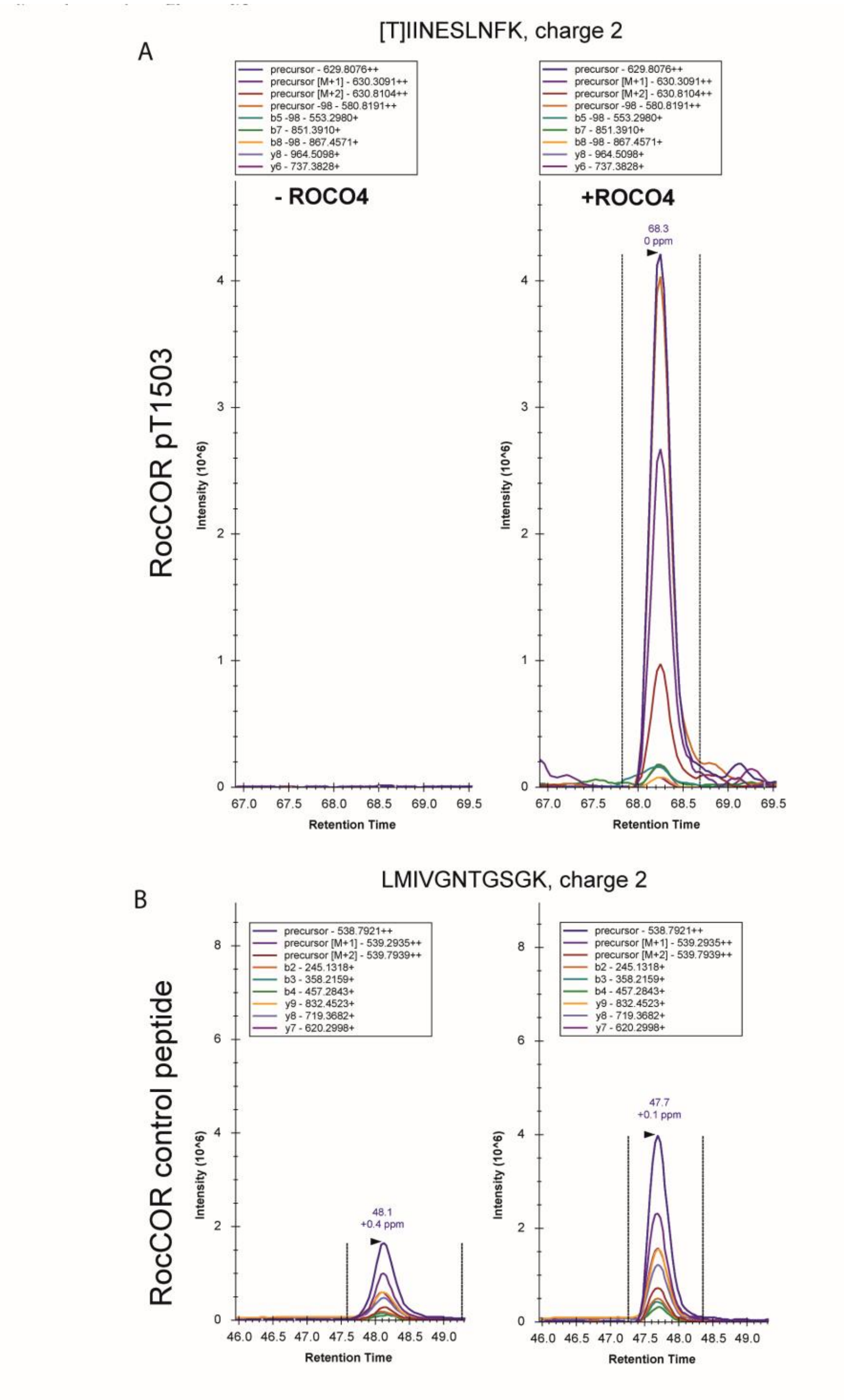

Supplementary Fig 2: Evidence for LRRK2 pT1503. (A) Extracted PRM spectrum for pT1503 for LRRK2 either incubated without or with ROCO4. (B) Extracted PRM spectrum for a reference peptide corresponding to the LRRK2 Roc sequence. This targeted approach only allowed us to confidently extract the pT1503 from the DIA data.

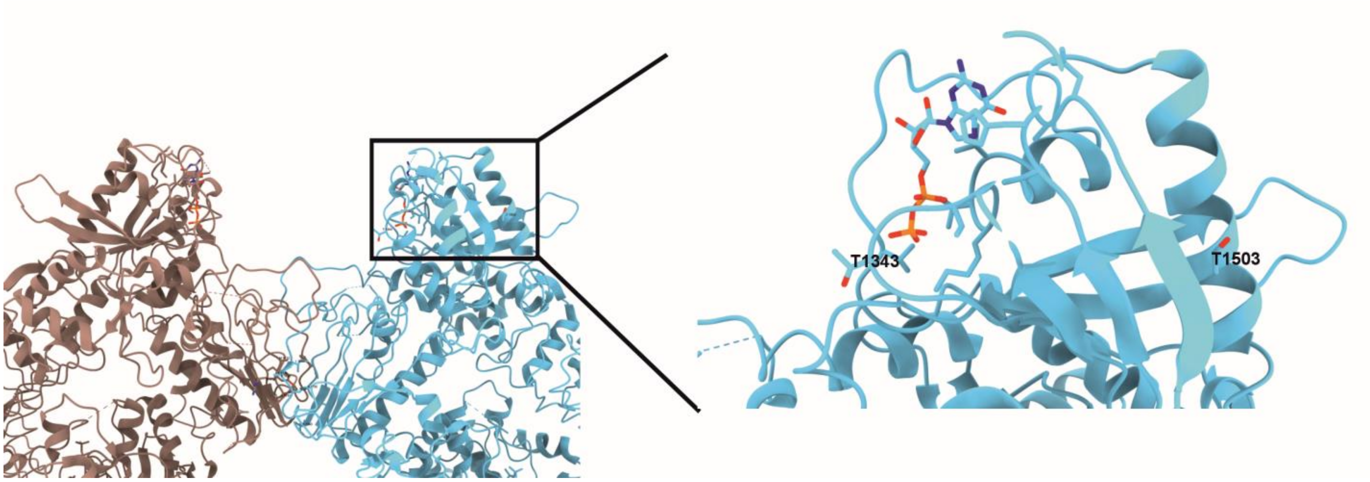

Supplementary Fig. 3: T1503 is in close proximity to the nucleotide-binding pocket of LRRK2. Left: Structure of LRRK2 dimer focusing on the RocCOR dimerization. The two protomers colored brown and blue respectively. Centrally, the dimerization interface can be seen, while on the top right (enlarged) the nucleotide-binding pocket (the T1343 and T1503 residues are found in close proximity to each other and the nucleotide binding pocket. T1343 has been previously shown to affect dimerization and kinase activity of LRRK2 (Gilsbach et al. 2024). PDB code used (7LHT) Myasnikov et al. 2021, Visualised in ChimeraX v. 1.10.

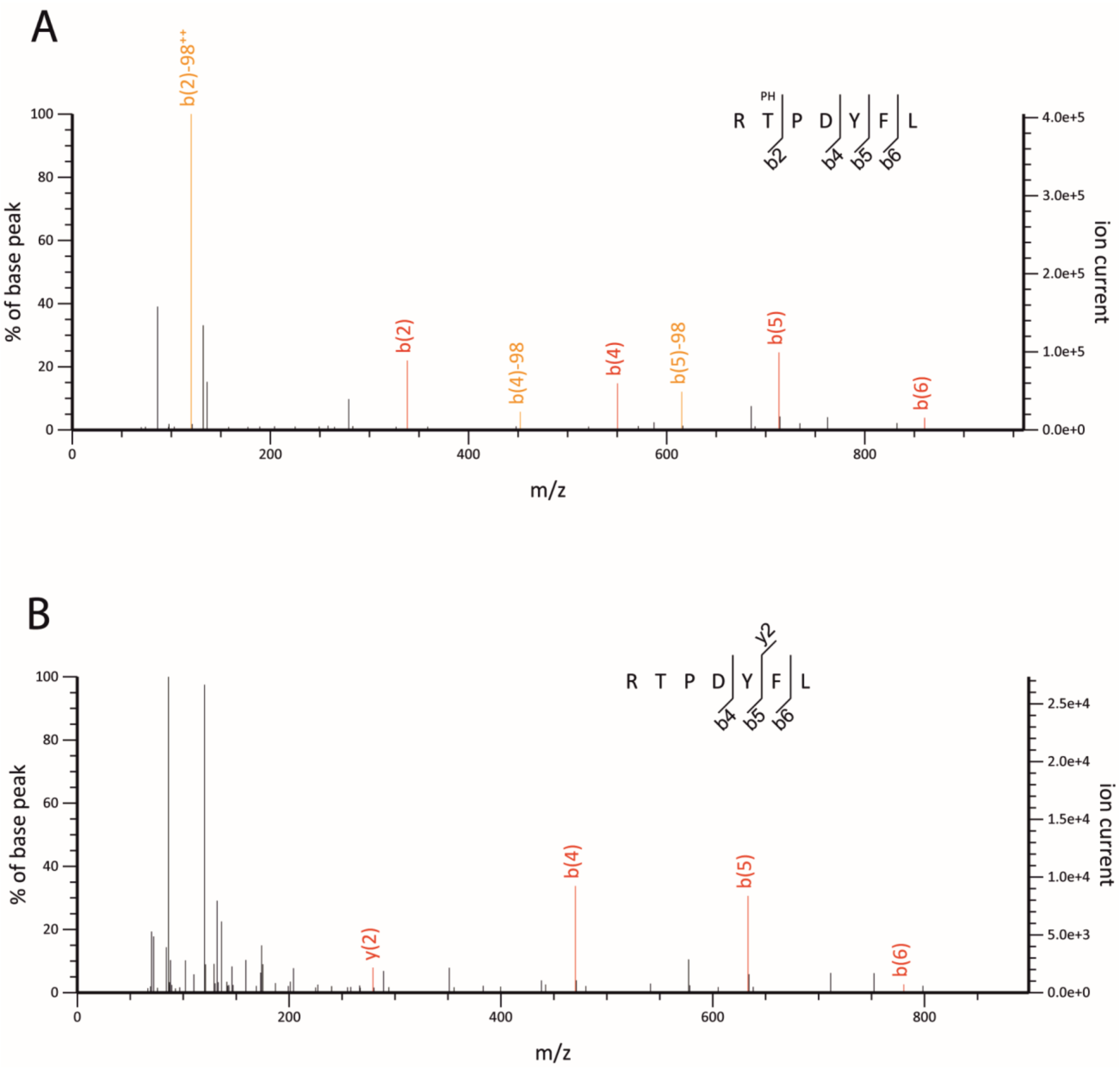

Supplementary Fig. 4: Evidence for the PPP2CA T304 phosphorylation. Mascot-annotated MS2 **s**pectra either for (A) the phosphorylated peptide or (B) unphosphorylated peptide are shown.

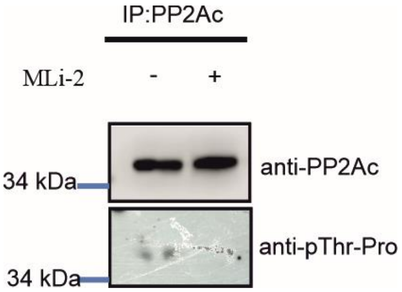

Supplementary Fig. 5: LRRK2 is able to dephosphorylate PPP2CA at T304 residue in A549 cells. A549 lung carcinoma cells were treated with 10nM MLi-2 LRRK2 kinase inhibitor for 90 minutes, prior to cell lysis. Furthermore, the PP2A catalytic subunits were immunoprecipitated. In the absence of the MLi-2 inhibitor, T304 signal of PP2A catalytic subunits is detected, however when the LRRK2 kinase activity is inhibited pharmacologically (by MLI-2), the pT304 signal is diminished.

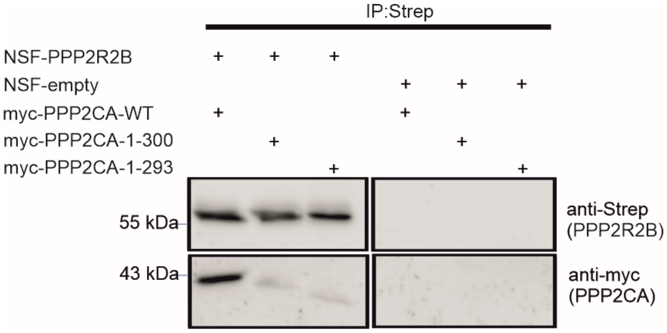

Supplementary Fig. 6: The c-terminal part of PPP2CA is essential for interaction with PPP2R2B subunit. NSF-PPP2R2B or NSF-empty and the indicated myc-tagged PPP2CA constructs were co-transfected in HEK293 cells. Subsequently the cells were lysed and streptavidin beads were used to pull down NSF- PPP2R2B. In addition, we checked whether myc-PPP2CA constructs are in the same complex with NSF- PPP2R2B. While myc-WT-PPP2CA interacts with NSF- PPP2R2B the myc-1-300aa-PPP2CA and myc-1-293aa-PPP2CA proteins are showing a very low interaction affinity with the NSF-PPP2R2B.
